# Use of α-Cyclodextrin to Produce Nanoencapsulated Curcumin to Preserve Curcumin Stability from Photooxidation While Simultaneously Enhancing Caco-2 Intestinal Uptake and Antioxidant Capacity

**DOI:** 10.3390/antiox15070901

**Published:** 2026-07-21

**Authors:** David D. Kitts, Yigong Guo, Maidinai Sabier, Alexandra Lizares, Anubhav Pratap-Singh, Anika Singh

**Affiliations:** 1 Food, Nutrition and Health, Faculty of Land and Food Systems, The University of British Columbia, Vancouver, BC V6T 1Z4, Canada; yigong_guo@bcit.ca (Y.G.); msabier@student.ubc.ca (M.S.); anubhav.singh@ubc.ca (A.P.-S.); 2 Natural Health and Food Products Research Group, Centre for Applied Research and Innovation (CARI), British Columbia Institute of Technology, 4355 Mathissi Pl, Burnaby, BC V5G 4S8, Canada; amtlizares@gmail.com

**Keywords:** curcumin, encapsulation, α-cyclodextrin, photooxidation, cell redox, TEER value

## Abstract

Curcumin is a major bioactive component present in turmeric, attributed with a bright yellow color; however, with low solubility and susceptibility to photooxidation, many of the purported health benefits are actually limited. We developed an encapsulation delivery system to produce curcumin nanoparticles using α-cyclodextrin as a carrier and Tween 80 as a surfactant for the purpose of increasing photostability and bioavailability. We used RSM to determine an optimal curcumin nanoparticle (NP) size of 386.4 ± 21.3 nm and entrapment efficiency of 97.4 ± 2.9% for use in functional tests conducted in differentiated Caco-2 cells. Curcumin-NP showed greater (*p* = 0.001) (74.32 ± 4.20%) stability compared to free curcumin (36.74 ± 3.03%), after treatment with pulsed light, at the highest 20 J/cm^2^ energy input. The release behavior of curcumin-NPs was pH-dependent and showed a 5 times higher intestinal epithelial cellular uptake and 21.3% decrease of TEER-value, compared to free curcumin (*p* = 0.017). Compared with free curcumin, the curcumin-NPs had higher antioxidant capacity in all tests while exhibited lower antioxidant capacity loss after pulsed light treatment. Moreover, pre-exposure of curcumin-NPs to pulsed light treatment showed no apparent cytotoxicity effects in Caco-2 cells. In conclusion, using α-cyclodextrin for curcumin-NP encapsulation enhanced both bioavailability and photostability, leading to greater intestinal intracellular antioxidant capacity and no loss in cell viability.

## 1. Introduction

Curcumin, 1,7-bis(4-hydroxy-3-methoxyphenyl)-1,6-heptadiene-3,5-dione, is a dominant curcuminoid polyphenol responsible for the yellow pigment and highly biologically active component in turmeric [1,2]. The linear diarylheptanoid structure of curcumin features essential hydroxyl and methoxy groups, which confer both antioxidant and antiproliferative activities, representing the underlying mechanisms for therapeutic applications related to disease prevention [3,4,5]. Despite a promising therapeutic potential, the clinical procedures that rely on curcumin efficacy are significantly limited by inherent physicochemical properties that include poor aqueous solubility at physiological pH [6] and rapid degradation [7], such as a susceptibility to undergo rapid photodegradation [8]. Efforts to improve curcumin bioavailability due to a relatively low solubility and stability have changed from focusing on the ionic structure of curcumin, whether being in a keto-enol equilibrium of full keto-form [9], to optimizing encapsulation innovation methods such as using various coacervation and nanoprecipitation techniques and different carriers that include nanoparticles, nanogels, nanospheres, emulsions, and micelles [10,11].

Cyclodextrins are cyclic oligosaccharides with a unique chemical structure that features an inner hydrophobic cavity and an outer hydrophilic surface. This structure enables cyclodextrins to act as solubilizing agents for water-insoluble substrates. There are numerous cyclodextrin derivatives, including alpha-, beta-, and gamma-cyclodextrins, used successfully to form inclusion complexes that support encapsulating hydrophobic molecules, such as curcumin [12,13,14], while also eliciting a low toxicity potential. In general, the use of cyclodextrins for microencapsulation strategies for curcumin is based on external factors, including enhanced stability and aqueous solubility, which improves overall dispersion and cellular uptake, thus potentially improving bioavailability [15]. Recent studies conducted with ß-cyclodextrin to encapsulate curcumin and other major curcuminoids reported positive results in enhancing stability and bioavailability [16]. Among all modified cyclodextrins, α-cyclodextrin is often favored over β-cyclodextrin for microencapsulation due to relatively higher water solubility and specific binding affinity for fat-soluble materials [17]. The α-cyclodextrin has a smaller cavity size compared with β-cyclodextrin, which can result in stronger host–guest interactions with small molecules like curcumin, thus enhancing encapsulation efficiency and stability. Novel intestinal-targeted controlled release capsules have recently been reported using a gelatin/sodium carboxymethyl cellulose in a complex coacervation and double emulsification method to optimize encapsulation efficiency as well as gastrointestinal controlled curcumin release [11]. However, these microscale delivery systems generally exhibit relatively large particle sizes, which may limit cellular uptake, aqueous dispersibility, and applications requiring nanoscale delivery. Therefore, developing α-cyclodextrin-based nano-sized inclusion complexes represents an alternative strategy that not only enhances curcumin solubility through host–guest interactions, but also offers improved colloidal stability and greater potential for food and nutraceutical applications.

While curcumin encapsulation using proteins, polysaccharides, and lipid nanoparticles has been extensively studied, there is less information specifically on the biological functionality of curcumin when formulated for nanoencapsulation using α-cyclodextrin as encapsulation material. In particular, there are no reports on how α-cyclodextrin can protect curcumin from photodegradation under pulsed light (PL) exposure, which uses short-duration flashes of broad-spectrum (UV to IR) light to create reactive oxygen species (ROS) and photochemical effects on exposed compounds. Pulsed light (PL) irradiation triggers ROS-mediated oxidative damage to polyphenols through a sequence of photochemical and pro-oxidant reactions. The high-intensity, broad-spectrum light (UV to near-infrared) generates reactive oxygen species (ROS) like singlet oxygen and hydroxyl radicals, which rapidly oxidize the polyphenols, degrading their structural integrity. This study aimed to optimize an encapsulation process for curcumin using nanoparticles (NPs) constructed with α-cyclodextrin, while also using Tween 80 to serve as a surfactant required to improve the solubility of lipophilic compounds needed to enhance bioavailability. The central hypothesis of this study is that encapsulation of curcumin within α-cyclodextrin inclusion complexes will enhance its stability and preserve functionality when exposed to pulsed light treatment. Stability and functionality of curcumin-NPs were assessed using 21-day-old Caco-2 intestinal cells, with vital factors focused on maximizing curcumin bioavailability, antioxidant capacity, and effects on cell viability being the primary objectives.

## 2. Materials and Methods

### 2.1. Materials

The commercial curcumin used in this study was purchased from Organika (Vancouver, BC, Canada), which contains approximately 77% curcumin, 17% demethoxycurcumin, and 6% bismethoxycurcumin [18]. Pure curcumin standard (>98%) was obtained from Sigma-Aldrich (Oakville, ON, Canada). α-cyclodextrin used in this study came from Tokyo Chemical Industry (Tokyo, Japan). All chemicals used in antioxidant tests (ORAC and ABTS) were ordered from Sigma-Aldrich (Oakville, ON, Canada). The human colon adenocarcinoma cell line was Caco-2 (HTB-37, American Type Culture Collection, Manassas, VA, USA). All other reagents used in this study were of analytical grade or HPLC grade.

### 2.2. Preparation and Optimization of Curcumin-NPs

A central composite design was used to optimize the curcumin-α-cyclodextrin mixtures (Minitab software version 18.0; Minitab LLC, State College, PA, USA), and results are presented in Table S1. Prior to conducting the optimization study, preliminary experiments identified the key parameters affecting nanoparticle characteristics. Based on these trials and the previous literature, α-cyclodextrin concentration and curcumin to α-cyclodextrin and Tween ratio were selected as the main factors for further optimization. The curcumin nanoparticles (NPs) were prepared using a two-step homogenization method, where curcumin was dissolved in ethanol (e.g., 1 mg/mL) and α-cyclodextrin dissolved in DD water, to reach a final test curcumin: α-cyclodextrin ratio. Tween 80 was added to the combined solution, and all contents were homogenized for 1 h using a polytron PCU-2-110 homogenizer (Brinkmann Ind., Westbury, NY, USA) under high-speed stirring at 12,500 rpm. The two variable factors used in the study design were the ratios of both curcumin and α-cyclodextrin (*w*/*w*) and the curcumin and Tween 80 (*w*/*w*) (Table 1). Particle size and entrapment efficiency (EE) were used as two responses and were fitted to a non-linear model as shown in Equation (1).

Y = *β*_0_ + *β*_1_A + *β*_2_B + *β*_1_^2^A^2^ + *β*_2_^2^B^2^ + *β*_1_
*β*_2_AB (1)

where Y equals the response variable, *β*_0_ = the intercept term, *β*_1_–*β*_2_ = linear coefficients, *β*_1_ *β*_2_ = interactive coefficients, *β*_1_^2^–*β*_2_^2^ = quadratic coefficients, and A and B represent the dependent response variables (particle size and EE, respectively).

The final optimized NPs ready for use were dehydrated by spray drying to remove ethanol residue. The parameters were set up at inlet 90 °C, outlet 40 °C, aspirator 100%, feed 1 mL/min, and air flow 470–740 L/h.

### 2.3. Characterization of the Curcumin-NPs

Curcumin-NP particle size and PDI were determined using a Litesizer 500 dynamic light scanner (DLS) (Anton Paar, Graz, Austria). Cross-linking established between α-cyclodextrin and curcumin-NPs was measured using Fourier Transform Infrared-Attenuated total reflectance (FTIR-ATR) spectroscopy. Free curcumin, α-cyclodextrin, the physical mixture of free curcumin and α-cyclodextrin, and optimized curcumin-NPs were measured using a Spectrum 100 FTIR spectrophotometer (PerkinElmer, Waltham, MA, USA) under a spectral range of 600–4000 cm^−1^ at a resolution of 4 cm^−1^. Baseline corrections were applied to all acquired FTIR spectra. A region was selected in the spectra that had the highest degree of variation through mid-infrared range. This region was isolated and transformation was used to enhance the FTIR signal and suppress unwanted instrument background features or sample matrix composition [19]. Morphology of optimized curcumin-NPs was assessed using a Hitachi H7600 Transmission electron microscopy (Hitachi, Tokyo, Japan). The entrapment efficiency (EE) of the curcumin-NPs was determined using an Agilent 1260 series HPLC system (Agilent, Santa Clara, CA, USA) and calculated according to Equation (2). Unencapsulated curcumin was removed using ultrafiltration with a 10 kDa molecular weight cutoff. The total curcumin content in the curcumin-NPs and unencapsulated curcumin were determined by HPLC, using a C18 column (Zorbax, 3.5 μm, 4.6 mm × 150 mm, Agilent, USA), and a mobile phase of acetonitrile and water with 0.1% TFA in a gradient ratio from 10/90 to 100/0 for 10 min run under 425 nm [18].

Equation (2)


EE (%) = 1 − ((Unencapsulated curcumin)/(Total curcumin)) × 100%
(2)

### 2.4. Pulsed Light Treatment and Curcumin Retention Test

Free and optimized curcumin-NPs were both treated with PL. Free curcumin and optimized curcumin-NP powder containing the same curcumin content (100 mg) were placed under the center of a PL lamp in a glass container that was positioned 5 cm from the light source, and the temperature was carefully controlled. The fluence per pulse, determined using a radiometer, was set at 0.016 J/cm^2^, and total fluence (related to both frequency and duration time of PL) was applied at 5 J/cm^2^, 10 J/cm^2^, 15 J/cm^2^, and 20 J/cm^2^ for all samples. The determination of curcumin retained after the PL treatments was measured by HPLC as described above. The pure curcumin standard was used to quantify the retention of the curcumin after PL treatment.

### 2.5. In Vitro Release Behavior of Optimized Curcumin-NPs in Simulated Gastrointestinal Conditions

The release behavior of optimized curcumin-NPs was determined using a dialysis bag method with a molecular cutoff of 10 kDa, at pH values that were to designed to simulate gastric (pepsin at pH 2.4), proximal duodenum (pancreatin at pH 6.5), and distal ileum small intestine (pancreatin and bile salts at pH 7.1) conditions with continuous shaking at 150 rpm for 6 h. Recovery of curcumin released from the curcumin-NPs was analyzed by HPLC. Release rates of curcumin samples were calculated from the ratio of released to total curcumin measured in test samples.

### 2.6. Antioxidant Assay of the Curcumin and Curcumin-NPs Treated with PL

The antioxidant capacity of both free curcumin and curcumin-NPs treated with PL was first tested using standard chemical ABTS and ORAC assays, with Trolox representing the antioxidant standard. The ABTS•+ working solution consisted of reacting ABTS (7 mM) with potassium persulfate overnight, which when diluted gives an absorbance of 0.70 ± 0.02 at 734 nm using a Tecan infinite M200 pro spectrophotometer plate reader (Tecan, Männedorf, Switzerland). The radical scavenging capacity of samples was calculated according to Equation (3):

(3) $$\mathrm{ABTS}•+\;\mathrm{scavenging}\;=\;(\frac{Absorbance\;of\;working\;solution-Absorbance\;of\;sample/trolox}{Absorbance\;of\;working\;solution})\times 100\%\;$$

The ORAC assay consisted of samples and Trolox standards (e.g., 10–40 uM in phosphate buffer) mixed with 200 nM of FSS in black 96-well plates Sigma-Aldrich (Oakville, ON, Canada). Freshly prepared AAPH (60 nM) was added to samples as the peroxyl radical inducer, and samples were incubated for 10 min. The negative control consisted of the phosphate buffer along with 200 nM of FSS in phosphate buffer. Fluorescence readings at excitation of 485 nm, with emission of 527 nm, was recorded each minute for one hour using a Tecan infinite M200 pro spectrophotometer plate reader (Tecan, Männedorf, Switzerland). The areas under the Trolox curve (AUC) and unknown curcumin samples were plotted against concentration, and ORAC antioxidant values were calculated from the ratio regression equation slopes for each sample and Trolox standard, respectively (Equations (4) and (5)). ORAC antioxidant capacities of curcumin samples were expressed as μmol Trolox Equivalent/μmol sample.

AUC = ∑_(*n* = 1)^60 f_*n*/f_1 = 1 + f_2/f_1 + f_3/f_1 +⋯+ f_59/f_1 + f_60/f_1 (4)

where f*_n_* is the fluorescence intensity at time *n* [20].

The AUC was plotted against concentration of Trolox (standard) or sample, which yielded a regression equation for each sample or Trolox. The slope of the linear regression was used to determine the antioxidant capacity, expressed as ORAC value.


ORAC value = ((Slope sample)/(Slope Trolox)) × 100%
(5)

### 2.7. Caco-2 Cell Culture Conditions

Caco-2 cells were seeded in 96-well clear tissue culture-treated microplates at a density of 1 × 10^5^ cells/cm^2^ (3.2 × 10^5^ cells/mL, 100 μL per well) and cultured in Dulbecco’s Modified Eagle’s Medium (DMEM), supplemented with 10% fetal-bovine serum (FBS), 100 U/mL penicillin, and 100 U/mL streptomycin in a humidified atmosphere containing 5% CO_2_. To establish epithelial monolayer formation and cell differentiation, the culture medium was refreshed every 2–3 days, and cells were sub-cultured at approximately 80% confluence cells for 21 days. For all assays, cells used had a passage of 25–28.

### 2.8. In Vitro Cell Uptake and Transepithelial Electric Resistance (TEER Value) of Curcumin from Curcumin-NPs

The Caco-2 cell uptake of free curcumin and curcumin-NPs was tested in vitro using 21-day differentiated cells cultured at a density of 5 × 10^4^ cells/cm^2^ using 24-well plates with Corning^®^ inserts (Transwell pore diameter 0.4 μm, growth area 0.33 cm^2^) at 37 °C with 5% CO_2_ to avoid excessive multilayer formation while ensuring sufficient cell coverage for quantification. The TEER value in each well was tested for at least 250 Ω/cm^2^ for confirmation of formation of Caco-2 monolayer by a Millicell^®^-Electrical Resistance System. Sample treatments for the uptake study were done for 0, 0.5, 1, 2, 4, and 6 h after PL treatments for free curcumin, encapsulated curcumin-NPs, and both free and encapsulated curcumin samples. All samples were diluted to have a curcumin content of 1 mg/mL. Curcumin uptake was determined by sampling 100 ul of culture fluid from the basolateral side (with refill each time) and quantified by HPLC. Caco-2 cell TEER values were also determined for a subsample of the curcumin and curcumin-NPs that were exposed to 20 j/cm^2^ PL. Briefly, Caco-2 cells with a density of 2.5 × 10^5^/cm^2^ were seeded in 24-well translucent polyethylene terephthalate membrane transwell inserts (0.4 µm) (BD Biosciences, San Jose, CA, USA) to facilitate the formation of confluent, differentiated monolayers with stable barrier properties. They were grown in DMEM medium for 21 days to reach differentiation. Transepithelial electrical resistance (TEER) values were measured using a volt-ohmmeter (Millicell^®^ ERS, Millipore, Bedford, MA, USA). TEER values were measured three times on each well. A relative epithelial resistance (%) was calculated according to the initial value taken at T = 0 min [21]. TEER values were also measured after the experiment to confirm that barrier integrity was maintained.

### 2.9. In Vitro Caco-2 Antioxidant Study of Free Curcumin and Curcumin-NPs Treated with PL

The relative affinity of both control and pulsed light-treated samples to mitigate peroxyl radical-induced intracellular oxidative stress in Caco-2 cells was assessed using the 2′,7′-dichlorodihydrofluorescein diacetate (DCFH-DA) assay, with modifications to the method described before [18,20]. Caco-2 cells were seeded into 96-well black microplates with clear bottoms at a density of 3.2 × 10^5^ cells/mL (100 μL per well) according to the requirements of the assay format to achieve consistent fluorescence measurements. They were cultured for 21 days in DMEM supplemented with 10% FBS, 100 U/mL penicillin, and 100 μg/mL streptomycin, under a humidified atmosphere of 5% CO_2_ at 37 °C. The culture medium was refreshed every 2–3 days. After 21 days, differentiated cells were washed twice with phosphate-buffered saline (PBS) and exposed to 50 μM curcumin samples (untreated or PL-treated for both free and curcumin-NPs) in serum-free DMEM for 24 h. This concentration was determined from a preliminary concentration-dependent experiment using untreated free curcumin (0–100 μM). Following the 24 h incubation, the samples were removed, and cells were washed twice with PBS. Subsequently, 5 μM DCFH-DA was added, and the cells were incubated for 30 min at 37 °C in the dark. After this incubation, 1 mM 2,2-azobis(2-amidinopropane) dihydrochloride (AAPH) was added. The cells were then continuously incubated for another hour, and fluorescence was measured (excitation: 485 nm, emission: 527 nm) using a Thermo Labsystems Multiskan Spectrum microplate spectrophotometer. Blank nanoparticles were also tested to eliminate the background noise. Data were expressed as the percentage of ROS inhibition, calculated using Equation (6):

ROS inhibition (%) = (fpc − fsample)/(fpc − fnc) × 100
(6)

where fpc, fsample, and fnc refer to the fluorescence intensities measured from cells treated with the positive control (both AAPH and DCFH-DA), fsample (AAPH and DCFH-DA), and negative control (DCFH-DA only), respectively.

### 2.10. Caco-2 Cell Viability Assay (MTT) Assay

Cellular metabolic activity was used as the indicator of Caco-2 cell viability after treatment with free curcumin and curcumin-NPs (50–1000 µM, according to a modified MTT 3-(4,5-dimethylthiazol-2-yl)-2,5-diphenyltetrazolium bromide assay) [20]. Cells were incubated with 100 μL of MTT solution (0.5 mg/mL in serum-free DMEM) for 6 h, allowing metabolically active cells to reduce MTT to formazan crystals. Subsequently, 100 μL of dimethyl sulfoxide (DMSO) was added to dissolve the formazan crystals prior to determining absorbance values (Abs = 570 nm) using a Thermo Labsystems Multiskan Spectrum microplate spectrophotometer. Cell viability was expressed as a percentage of the control, calculated using Equation (7):


Cell viability (%) = (Absorbance (570 nm) of the cells treated with sample)/(Absorbance (570 nm) of the negative control (media only)) × 100
(7)

### 2.11. Statistical Analysis

All experiments were performed in triplicate, and results are expressed as mean ± standard error (SEM). Normality and homogeneity of variance were assessed prior to analysis. Data were analyzed with one-way ANOVA, followed by Tukey’s post hoc test (*p <* 0.05) or paired *t*-test, using IBM SPSS Statistics 26 (IBM, Endicott, NY, USA).

## 3. Results and Discussion

### 3.1. Optimization of Curcumin-NPs

The results described by regression equations obtained from optimizing experiments using a 2-factor central composite design are given in Table S1, and were used as shown in Equations (7) and (8) to establish the optimal combination of nanoparticle size and entrapment efficiency from varying ratios of curcumin and α-cyclodextrin (A, *w*/*w*) and curcumin and Tween 80 (B, *w*/*w*), respectively.


Particle size = 491 − 6A − 55B + 18.7A2 + 16.9B2 − 24.7AB
(8)

EE = 65.17 + 12.08A + 6.60B − 1.174A2 − 0.961B2 − 0.567AB
(9)

**Figure 1:**
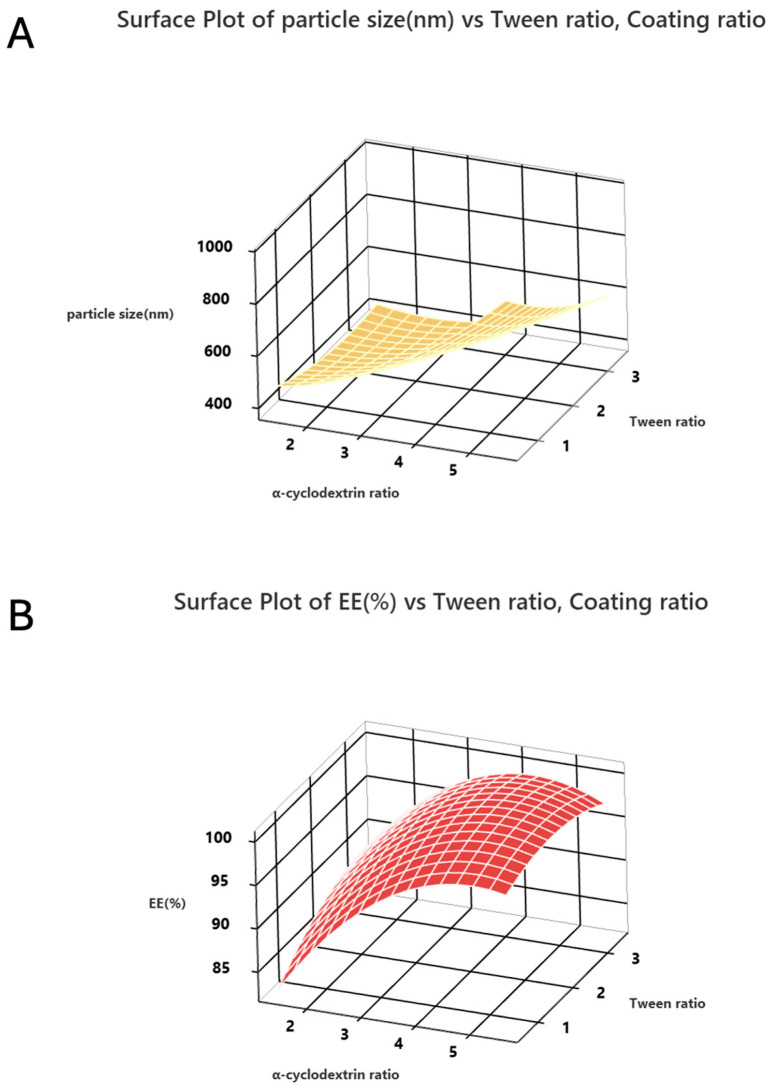
Three-dimensional response surface plots of RSM design for the relationship between (**A**) particle size, (**B**) entrapment efficiency (EE), the ratio of α-cyclodextrin, Tween, and curcumin, respectively.

From RSM analysis, based on the results of the RSM design, variables A and B were statistically significant toward modeling particle size, while variables A and A^2^ were statistically significant toward modeling EE. The coefficient of determination (R^2^), adjusted R^2^, and predicted R^2^ for the fitted models are shown in Table S3. The model exhibited a high coefficient of determination (R^2^ = 0.95 and 0.98), indicating that over 95% and 98% of the experimental variability in particle size and encapsulation efficiency was explained by the selected formulation variables, respectively. The lack-of-fit test was statistically insignificant (*p* > 0.05), confirming that the residual variation mainly originated from random experimental error rather than systematic deviation between the model and the experimental observations. Experimental validation was performed to verify this fitting model, with discrepancy below 5% as well. The advantage of using α-cyclodextrin to encapsulate curcumin was related to a sufficient capacity to form an inclusion complex, facilitated by the unique structure of having both a hydrophilic exterior and a hydrophobic interior cavity, which functioned to encapsulate curcumin and allow subsequent release. As such, we propose that the hydrophobic curcumin was retained in the cavity largely by non-covalent bonding with Tween 80, as the surfactant, thus enabling α-cyclodextrin to function as the coating material. The more α-cyclodextrin used for curcumin coating resulted in higher EE and bigger particles. In addition, with Tween 80 being the chosen surfactant, the surface tension between the curcumin particles and the surrounding medium was reduced, which allowed greater content with Tween 80 to produce smaller and more uniform particles. The use of 3D response plots, presented in Figure 1A, allows visualization of the interaction between α-cyclodextrin and Tween on the particle size, with increases in α-cyclodextrin ratio having parallel increases in particle size and increasing EE. The Tween 80 effect was found to impact only the size of particles, as shown by a higher ratio of Tween producing smaller curcumin particle sizes. Increasing the content of α-cyclodextrin resulted in relatively high encapsulation capacity and noticeable interactions (Figure 1B).

### 3.2. Characterization of the Optimized Curcumin-NPs

**Figure 2:**
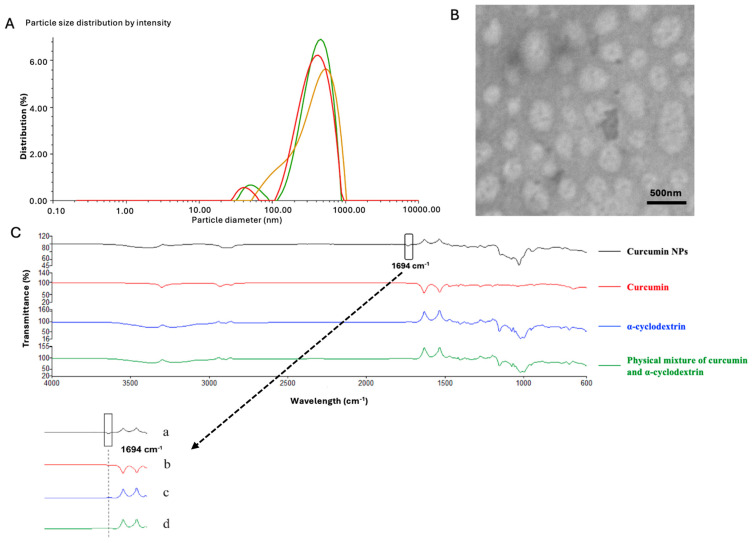
Characterization of the optimized curcumin-NPs: (**A**) = particle size distribution (in triplicate)(line colors are same as noted treatments in 2C); (**B**) = morphology of NPs (circular spherical structures with a uniform size) obtained by TEM; and (**C**) = FTIR spectra (validation = 1694 cm^−1^). Treatments: a = curcumin-NPs; b = free curcumin; c = α-cyclodextrin; d = physical mixture of curcumin and α-cyclodextrin.

The optimal conditions for preparing curcumin-NPs were visualized using both graphical and numerical optimization methods, whereby the ultimate goal was to establish the lowest particle size and the maximum entrapment efficiency. From the results presented, optimization was achieved at the combined levels of ratios of curcumin and α-cyclodextrin and Tween 80 to yield optimal particle size of 395.5 nm, with EE of 96.26% (Figure S1). The optimal formulation predicted by the model was experimentally validated by preparing three independent batches under the optimized conditions. The measured responses were compared with the predicted values to evaluate the accuracy of the model. The confirmation experiments resulted in particle size at 386.4 nm and EE at 97.4% (Figure 2A). No significant differences were identified between experimental and predicted values for all responses (error < 5.0%). Although the lack-of-fit test for particle size was significant (Table S1), the model still provided qualitative insight into the influence of formulation variables. Visualization of these results was done using TEM to observe particle morphology (Figure 2B). Together with its PDI of 0.21, it was confirmed that the optimized curcumin-NPs produced particles with spherical structures and a uniform size distribution. Results of additional validation of producing optimal curcumin-NP formations using FTIR are shown in Figure 2C. Compared with a physical mixture of curcumin and α-cyclodextrin, an increased peak occurring at 1694 cm^−1^ resulted from the curcumin benzene ring interaction with the electron-rich cavity of α-cyclodextrin (Figure 2C), leading to higher electron cloud density and a corresponding rise in this frequency [22]. In the curcumin-NPs, all sharp peaks corresponding to α-cyclodextrin remained, while most curcumin-related peaks were diminished in the FTIR [23]. These observations confirm the package of curcumin in α-cyclodextrin.

### 3.3. Curcumin Retention After PL Treatment in Free Curcumin and Curcumin-NPs

**Figure 3:**
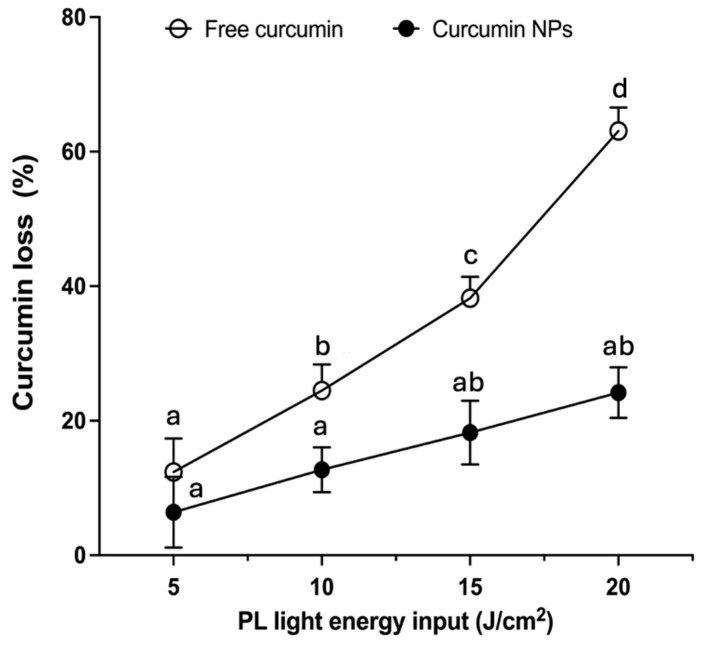
Curcumin reduction of the free curcumin (○) and curcumin-NPs (●) treated by PL (range = 5–20 J/cm^2^). Values are mean ± SEM (n = 3). a–d represent the significant difference between free curcumin and curcumin-NPs in same PL treatment group (*p* < 0.05).

Relative stability curves for curcumin, present in both free form and NP-encapsulation form following exposure over a wide range of PL treatments are shown in Figure 3. The retention of curcumin over different PL inputs of the NP-encapsulated form was compared to free curcumin. At the lowest PL energy (e.g., 5 J/cm^2^), retention of curcumin in free form was 89.42%; however, retention declined sharply to 36.74% at 20 J/cm^2^ PL treatment. These responses were comparatively inferior (*p* < 0.05) to the NP-encapsulated curcumin retentions of 93.43% receiving 5 J/cm^2^ PL, and 74.32% after 20 J/cm^2^ PL. Our former study showed the susceptibility of free curcumin degradation with PL exposure producing a number of curcumin dimers [18]. Herein, we extend this by showing that the formulation of curcumin-NPs constructed using α-cyclodextrin encapsulation was effective at preventing photooxidation induced by PL exposure to a notable level across all ranges of PL energy. This advantage of using curcumin-NP encapsulation to protect against photooxidation is likely attributed in part to the very high EE (e.g., 97.4%) established with the α-cyclodextrin core material when exposed to the wide range of PL energy. Moreover, compared with β-cyclodextrin, the α-cyclodextrin can better protects smaller compounds because of its tighter cavity fit, which minimizes the exposure of the encapsulated curcumin to external degrading factors [24].

### 3.4. Release Behavior of Curcumin-NPs

**Figure 4:**
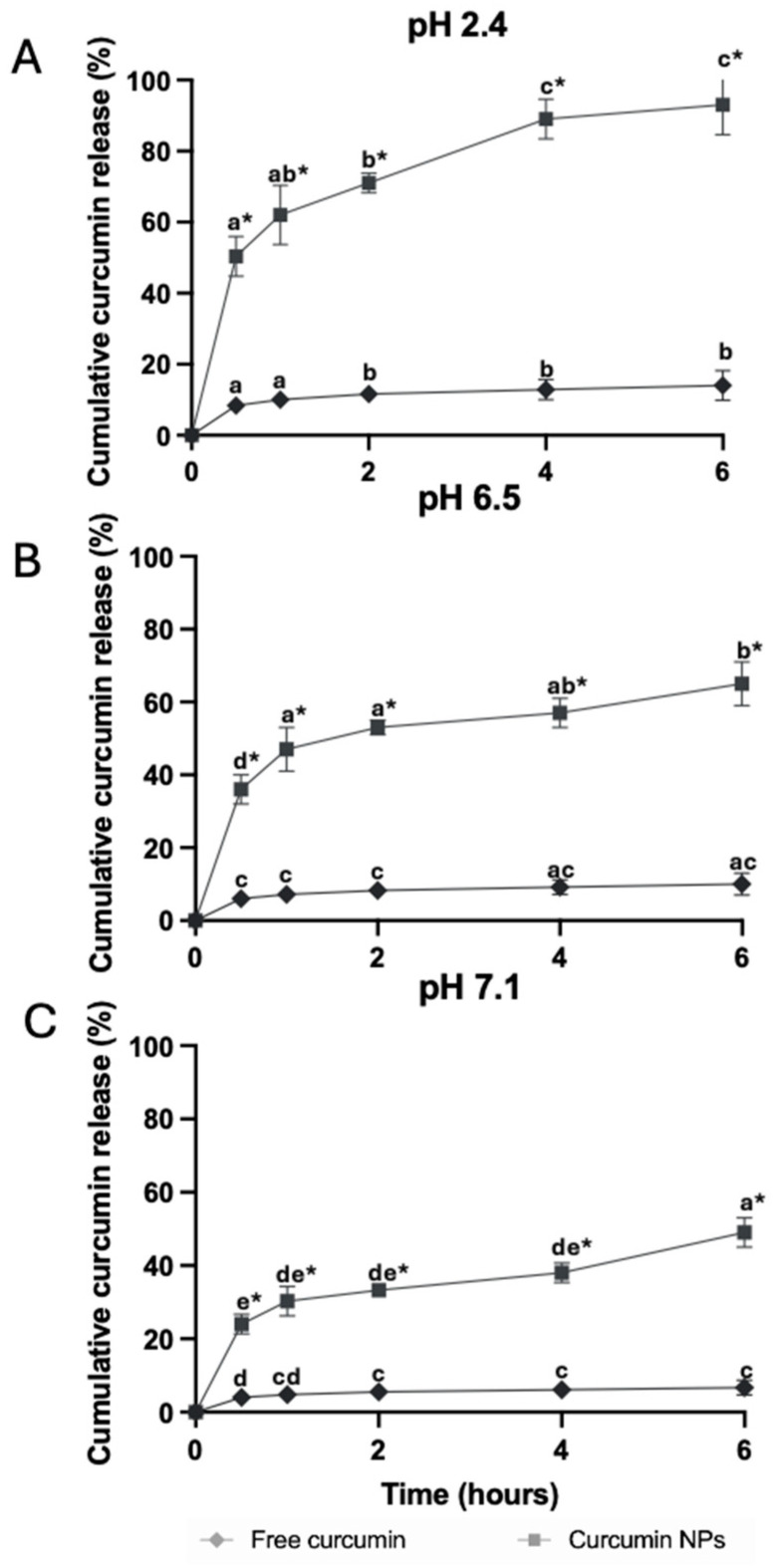
Release behavior of curcumin and curcumin-NPs under different pH conditions simulated for: (**A**) = stomach pH 2.4; (**B**) = small intestine pH 6.5; (**C**) = large intestine pH 7.1. Values represent means ± SEM (n = 3). Superscripts with different letters are significantly difference (*p* < 0.05) for free curcumin or curcumin-NPs in different pH treatments. * Represents significant difference between free curcumin and curcumin-NPs within the same pH treatment (*p* < 0.05).

The relative rate of curcumin released from encapsulated curcumin-NPs after 6 h incubation in PBS buffer, with pH values fixed at 2.4 (Figure 4A) stomach, 6.5 (Figure 4B) small intestine, and 7.1 (Figure 4C) large intestine were used to simulate the environment of the gastrointestinal tract (Figure 4). Curcumin release was minimal when in free form, over a wide range of pH values, thus showing the poor solubility throughout the gastrointestinal track that explains the low bioavailability reported by others [10]. This was contrasted with a much greater release of curcumin when presented in the optimized curcumin-NP formulation, which in turn was pH-dependent and followed first order kinetics. Curcumin release dropped from a maximum of 93% at pH 2.4, to 65% at pH 6.5, and 49% at 7.1 (*p* < 0.05) (Table S2). Under conditions typical for gastric pH, the retention of curcumin within the α-cyclodextrin core material would be expected to be relatively weak, since a combination of non-covalent and hydrophobic interactions within the α-cyclodextrin cavity would be susceptible to hydrolysis under acid conditions. Noticeably, the release study was designed to evaluate pH-dependent curcumin release under standardized experimental conditions rather than to fully simulate gastrointestinal digestion. Future studies are recommended to employ sequential simulated gastric and intestinal fluids containing pepsin, pancreatin, and bile salts to better mimic the physiological gastrointestinal environment.

### 3.5. Antioxidant Activity of Free Curcumin and Curcumin-NPs

**Figure 5:**
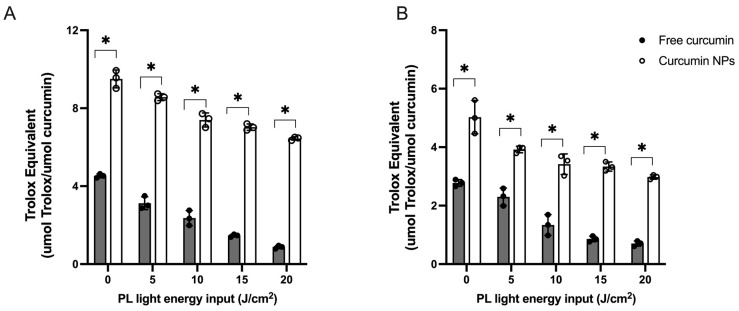
Chemical-based antioxidant capacity of free curcumin (●) and curcumin-NPs (○) treated with PL ranging from control to 20 J/cm^2^. (**A**) = ABTS and (**B**) = ORAC. Values represent mean ± SEM (n = 3). * Represents significant difference between free curcumin and curcumin-NPs within the same PL treatment group (*p* < 0.05).

A relative comparison of the antioxidant capacities determined using different chemical-based assays for free curcumin and the optimized curcumin-NPs following PL treatment at different energy levels is shown in Figure 5. Measures of antioxidant capacity, reported using both ABTS and ORAC, showed similarly relatively higher Trolox equivalents (*p* < 0.05) for curcumin-NPs compared to free curcumin, over a wide range of PL energies. Overall, PL treatment lowered the capacity of curcumin to quench ABTS^•+^ and peroxyl radicals from both free and encapsulated curcumin-NPs (*p* < 0.05); however, a consistent finding over a wide range of energies used in the experiment was the relatively lower reduction in activity with the curcumin-NPs (*p* < 0.05). This result confirms the protective effect of α-cyclodextrin on curcumin photooxidation when exposed to PL, which underlies the requirement to maintain the chemo-physical matrix of curcumin for related antioxidant activity. The reduced capacity for PL-treated curcumin is likely attributed to the formation of dimers, which alter the molecular structure of curcumin, namely, the available phenolic (–OH) groups, which are crucial for free radical quenching activity.

### 3.6. In Vitro Caco-2 Intracellular Uptake of Curcumin-NPs

**Figure 6:**
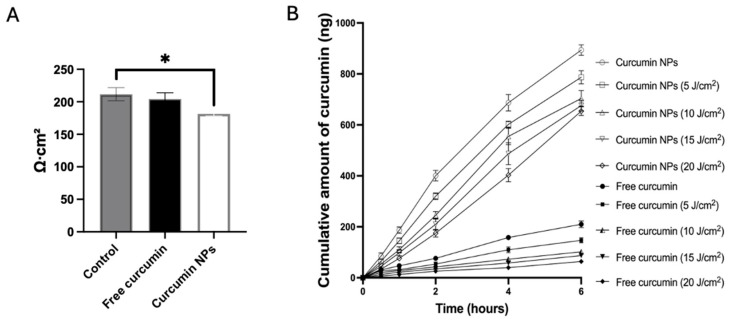
(**A**) = TEER values response in differentiated Caco-2 Cells. (**B**) = Intracellular free curcumin (●, ■, ▲, ▼, ◆) and curcumin-NP (○, ☐, △, ▽, ◇) uptake in Caco-2 cells, with prior PL treatments ranging from control to 20 J/cm^2^. Values represent mean ± SEM (n = 3). * Represents the significant difference between free curcumin and curcumin-NPs (*p* < 0.05).

Caco-2 monolayer cells were used to compare the relative cellular uptake of free curcumin compared to curcumin-NPs, and the impact of the prior PL treatments at different energy levels on curcumin bioavailability (Figure 6). Although our release study showed that 93% of the curcumin was released at gastric pH (2.4) after 6 h, in a real situation, most NPs stay in the stomach for less than 1 h so that lots of them can reach the intestine. Curcumin presented in the NP α-cyclodextrin encapsulation formulation showed markedly greater uptake (*p* < 0.05) compared to free curcumin, indicating that the nano-sized NPs were effectively dispersed to improve solubility, which facilitated the greater cell penetration compared to free curcumin. Similarly, applying PL over a wide range of energy levels, ranging from a very low dose (e.g., 5 J/cm^2^) to the highest (20 J/cm^2^) PL dose for a 6 h duration to free curcumin, dramatically reduced intracellular uptake by approximately 5-fold compared to the curcumin-NP formulation (*p* < 0.01). Despite the effect of PL treatment to lower Caco-2 cell uptake observed for both free curcumin and curcumin-NP samples, curcumin-NPs showed a relatively higher uptake over the complete range of PL treatments, which was consistent was our curcumin retention study. This can be attributed to the use of α-cyclodextrin as the coating material to encapsulate curcumin and the property that allowed interaction with the cell membrane lipid components to facilitate increased curcumin permeability by paracellular leakage [10]. Results of the cell permeability study support this explanation, as TEER values were decreased around 20% in the curcumin-NP group, while the free curcumin had no effect on Caco-2 intestinal TEER values (Figure 6A). It was reported that cyclodextrins can modify cell tight junctions primarily by displacing cholesterol from lipid rafts within the cell membrane, a feature considered critical for the structural integrity and function of membrane tight junctions. Cholesterol depletion proceeds loss of essential tight junction proteins, such as claudins and occluding, which function to facilitate paracellular absorption [25]. Recent work using cyclodextrin in Caco-2 cells showed that cholesterol extraction led to a significant decrease in TEER and increased paracellular transport, accompanied by the redistribution of tight junction proteins such as claudins and occluding [25]. The propensity of the α-cyclodextrin–curcumin-NP complex to permeate the cell would remove the barrier limiting uptake of the curcumin-NP. Moreover, as tight junction interpretation raises important safety concerns related to epithelial barrier integrity, a cell toxicity study was also conducted in following section. In this case, α-cyclodextrin was shown to be an excellent delivery vehicle for curcumin (Figure 6B), with the versatility to both improve solubility and capacity to also alter cell membranes to an extent that facilitated either passive or receptor-mediated uptake, resulting in greater amounts of curcumin penetrating Caco-2 cells.

### 3.7. In Vitro Intracellular Antioxidant Activity Curcumin-NPs

**Figure 7:**
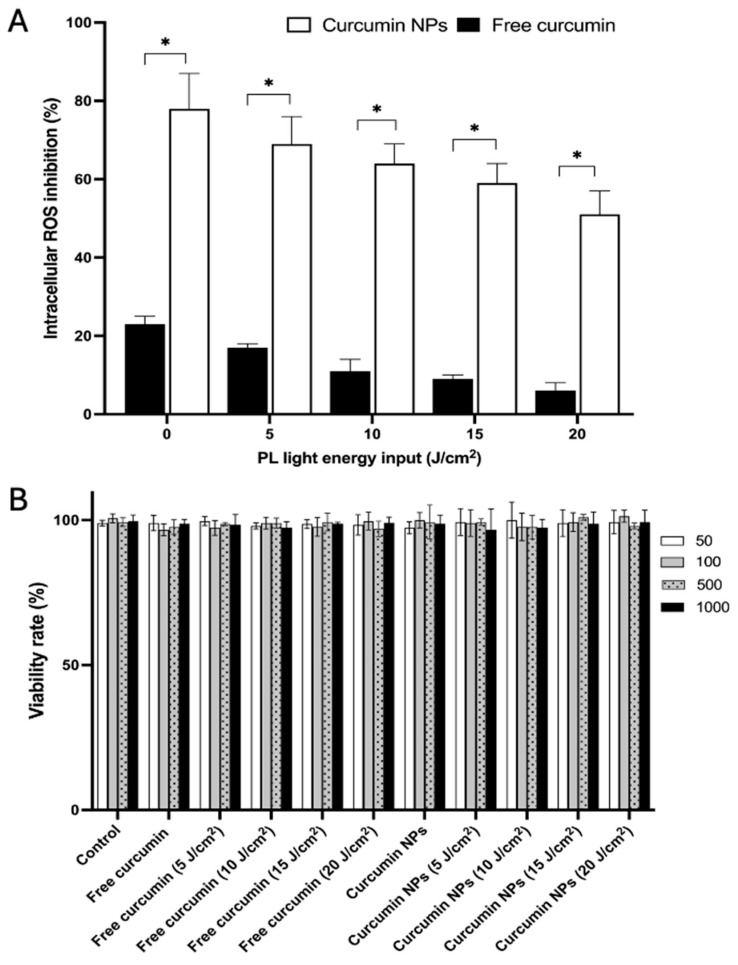
Caco-2 cell assays: (**A**) = intracellular antioxidant capacity of free curcumin (■) and curcumin-NPs (□) treated by PL ranging from control to 20 J/cm^2^; (**B**) = cell viability of differentiated Caco-2 cells treated by free curcumin and curcumin-NPs (concentration range = 50–1000 µM) treated by PL ranging from control to 20 J/cm^2^. Values represent means ± SEM (n = 3). * Represents the significant difference between free curcumin and curcumin-NPs in the same PL treatment group (*p* < 0.05).

The efficacy of curcumin and curcumin-NPs to mitigate oxidative stress in differentiated Caco-2 cells exposed to a peroxyl radical inducer is shown in Figure 7A. Intracellular antioxidant capacity enhancement using the DCFH-DA assay in Caco-2 epithelial cells confirmed the findings obtained using chemical ABTS and ORAC assays. Although free curcumin displayed significant free radical scavenging activity, the encapsulated curcumin-NP formulation produced superior activity that was 50% more effective (*p* < 0.05). For both free curcumin and curcumin-NPs, exposure to a range of PL treatments decreased antioxidant capacity (*p* < 0.05); however, this effect was more pronounced in the free curcumin compared to encapsulated curcumin-NPs. Our previous study reported that curcumin was effective in controlling the Caco-2 cellular redox and reduce oxidative stress by both scavenging intracellular free radicals and retaining cellular GSH levels when exposed to PL treatments that were the same magnitude used in non-thermal food processing [18]. Noticeably, this result was also related to the cell uptake, since more uptake can cause more control of the Caco-2 cellular redox and reduce oxidative stress. In this study, α-cyclodextrin encapsulation was shown to be more effective at protecting curcumin from pulsed light oxidation at all energy levels tested, compared to free curcumin. A complement to these results was the finding that no cytotoxicity attributed to the PL treatment of curcumin-NPs occurred (Figure 7B). This finding has importance to this study, as we can conclude that the encapsulated nano-sized curcumin, when held in α-cyclodextrin–Tween particles, shows improved stability and antioxidant functionality, while also having no potential adverse effect on Caco-2 cell viability, potentially originating from oxidation products derived from curcumin dimers after treatment with high PL energy.

## 4. Conclusions

This study demonstrated that α-cyclodextrin was an affective core material for producing curcumin-NPs, showing maximum stability from photooxidation, while also improving intestinal cellular uptake and antioxidant scavenging capacity. These functional attributes of curcumin-NPs were obtained using RSM to determine optimal curcumin-NP particle size and entrapment efficiency, using the best combination ratios of α-cyclodextrin and curcumin (*w*/*w*) and Tween 80 and curcumin, respectively. Curcumin-NP release patterns were pH-dependent, indicating that curcumin-NPs would be released under stomach acidity. Curcumin-NPs showed greater intracellular uptake in differentiated Caco-2 cells compared with free curcumin. This observation corresponded to paracellular leakage by lower intestinal TEER values and greater antioxidant capacity in intestinal cell-based assays compared to free curcumin over a wide range of PL energies. In summary, the use of the α-cyclodextrin–Tween combination reported herein is a novel approach used to construct curcumin nanoparticles for improved solubility and bioavailability, while also being effective at protecting against photooxidation and enhanced related antioxidant capacity. These findings show promise in applications where α-cyclodextrin–Tween constructed curcumin-NPs can be formulated to protect against oxidative stress in vulnerable consumers. Despite the promising results, several limitations should be acknowledged. The in vitro release study was conducted using a simplified pH-based model, which may not fully reflect physiological gastrointestinal conditions. Furthermore, although the optimized encapsulation formulation demonstrated improved performance, additional in vivo study under more physiologically relevant conditions is needed to confirm applicability.

## Figures and Tables

**Table 1:** Preparing optimization conditions and responses obtained from the central composite design (CCD).

Std Order	Run Order	Pt Type	Blocks	*α*-Cyclodextrin Ratio	Tween Ratio	Particle Size (nm)	EE (%)
5	1	−1	1	1.37868	2	455.3	87.2
4	2	1	1	5	3	632.6	98.4
2	3	1	1	5	1	876.3	99.7
7	4	−1	1	3.5	0.58579	568.4	94.3
1	5	1	1	2	1	453.4	90.2
13	6	0	1	3.5	2	486.4	96.7
6	7	−1	1	5.62132	2	601.3	99.3
9	8	0	1	3.5	2	486.9	98.9
8	9	−1	1	3.5	3.41421	387.3	99.1
10	10	0	1	3.5	2	478	99.3
11	11	0	1	3.5	2	477.6	99
12	12	0	1	3.5	2	484.1	99.2
3	13	1	1	2	3	358.1	92.3

## Data Availability

The experimental data supporting the findings of this study are available from the corresponding author upon reasonable request.

## Supplementary Materials

The following supporting information can be downloaded at: https://www.mdpi.com/article/10.3390/antiox15070901/s1, Figure S1: Expected optimized curcumin NPs results given by Minitab; Table S1: Validation of paratmeters for modelling optimal particle size and entrapment efficiency using single point design; Table S2: First-order regression parameters describing curcumin release under different pH conditions; Table S3: The coefficient of determination (R^2^), adjusted R^2^, and predicted R^2^ for the fitted models.
