# Supplementary material for: Use of α-Cyclodextrin to Produce Nanoencapsulated Curcumin to Preserve Curcumin Stability from Photooxidation While Simultaneously Enhancing Caco-2 Intestinal Uptake and Antioxidant Capacity
Source: Antioxidants (Basel). 2026 Jul 21;15(7):901. doi: 10.3390/antiox15070901 (PMC13404799; doi:10.3390/antiox15070901)
Supplement: Supplementary file 1 [file antioxidants-15-00901-s001.zip › antioxidants-4414764-supplementary.pdf]

Use of α-cyclodextrin to produce nanoencapsulataed curcumin that preserves curcumin stability from photooxidation while simultaneously enhancing Caco-2 intestinal uptake and antioxidant capacity

David D. Kitts[a*], Yigong Guo[ab], Maidinai Sabier[a], Alexandra Lizares[b], Anubhav-Pratap Singh[a] and Anika Singh[ab*].

[a] *Food, Nutrition and Health, Faculty of Land and Food Systems, The University of British Columbia, Vancouver, BC V6T 1Z4, Canada*

[b] *Natural Health and Food Products Research Group, Centre for Applied Research and Innovation (CARI), British Columbia Institute of Technology, 4355 Mathissi Pl, Burnaby, BC V5G 4S8, Canada*

* Shared Corresponding authors

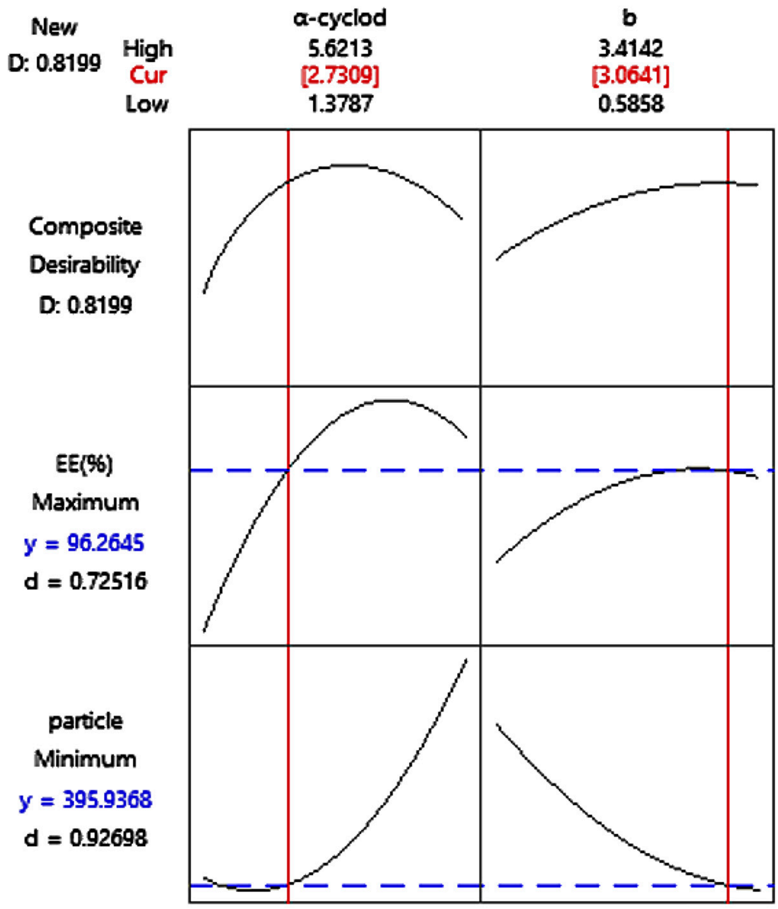

Figure S1. Expected optimized curcumin NPs results given by Minitab.

Table S1. Validation of paratmeters for modelling optimal particle size and entrapment efficiency using single point design.

Responses	Analysis of Variance					
A	Source	DF	Adj SS	Adj MS	*F*-value	*P*-value
	Model	5	165151	33030	5.39	0.024
	Linear	2	146394	73197	11.93	0.006
	a	1	102124	102124	16.65	0.005
	b	1	44270	44270	7.22	0.031
	Square	2	13251	6626	1.08	0.39
	a*a	1	12333	12333	2.01	0.199
	b*b	1	1982	1982	0.32	0.587
	Two-way Interaction	1	5506	5506	0.9	0.375
	a*b	1	5506	5506	0.9	0.375
	Error	7	42932	6133		
	Lack-of-Fit	3	42850	14283	702.41	0.089
	Pure Error	4	81	20		
	Total	12	208083			
B	Source	DF	Adj SS	Adj MS	*F*-value	*P*-value
	Model	5	194.69	38.938	28.58	0
	Linear	2	140.957	70.478	51.73	0
	a	1	133.759	133.759	98.17	0
	b	1	7.198	7.198	5.28	0.055
	Square	2	50.843	25.422	18.66	0.002
	a*a	1	48.53	48.53	35.62	0.001
	b*b	1	5.84	5.84	4.29	0.077
	Two-way Interaction	1	2.89	2.89	2.12	0.189
	a*b	1	2.89	2.89	2.12	0.189
	Error	7	9.537	1.362		
	Lack-of-Fit	3	4.829	1.61	1.37	0.373
	Pure Error	4	4.708	1.177		
	Total	12	204.228			

Note: The central point design involved 15 experimental trials. *A* = particle sizes; *B* = entrapment efficiency; *A*B* denote independent variable response interactions. The responses were fitted to a non-linear model, as described in Materials Methods.

Table S2. First-order regression parameters describing curcumin release under different pH conditions.

Different PH conditions	Sample type	a	b	R^2
2.5	FC	1.84	9.54	0.9873
	CN	17.88	42.74	0.9931
6.0	FC	1.42	6.31	0.9676
	CN	14.37	31.60	0.9744
7.0	FC	1.04	4.87	0.9674
	CN	12.91	22.64	0.9574

Note: FC, free curcumin; CN, curcumin nanoparticles (NPs). Curcumin release kinetics were fitted using the model: Released curcumin (t) = a·ln(t) + b, where a represents the release speed and b represents the release rate.

Table S3. The coefficient of determination (R^2), adjusted R^2, and predicted R^2 for the fitted models

Model summary	R-sq	R-sq(adj)	R-sq(pred)
Particle size	95.37%	84.63%	61.04%
Entrapment efficiency	98.33%	95.99%	82.58%
